# Modeling effects of voltage dependent properties of the cardiac muscarinic receptor on human sinus node function

**DOI:** 10.1371/journal.pcbi.1006438

**Published:** 2018-10-10

**Authors:** Robin Moss, Frank B. Sachse, Eloy G. Moreno-Galindo, Ricardo A. Navarro-Polanco, Martin Tristani-Firouzi, Gunnar Seemann

**Affiliations:** 1 Institute for Experimental Cardiovascular Medicine, University Heart Centre Freiburg/Bad Krozingen, Freiburg, Germany; 2 Faculty of Medicine, Albert-Ludwigs University of Freiburg, Freiburg, Germany; 3 Institute of Biomedical Engineering, Karlsruhe Institute of Technology (KIT), Karlsruhe, Germany; 4 Nora Eccles Harrison Cardiovascular Research and Training Institute, University of Utah, Salt Lake City, Utah, United States of America; 5 Biomedical Engineering, University of Utah, Salt Lake City, Utah, United States of America; 6 Centro Universitario de Investigaciones Biomédicas, Universidad de Colima, Colima, Mexico; Stanford University, UNITED STATES

## Abstract

The cardiac muscarinic receptor (M2R) regulates heart rate, in part, by modulating the acetylcholine (ACh) activated K^+^ current *I*_*K*,*ACh*_ through dissociation of G-proteins, that in turn activate K_ACh_ channels. Recently, M2Rs were noted to exhibit intrinsic voltage sensitivity, i.e. their affinity for ligands varies in a voltage dependent manner. The voltage sensitivity of M2R implies that the affinity for ACh (and thus the ACh effect) varies throughout the time course of a cardiac electrical cycle. The aim of this study was to investigate the contribution of M2R voltage sensitivity to the rate and shape of the human sinus node action potentials in physiological and pathophysiological conditions. We developed a Markovian model of the *I*_*K*,*ACh*_ modulation by voltage and integrated it into a computational model of human sinus node. We performed simulations with the integrated model varying ACh concentration and voltage sensitivity. Low ACh exerted a larger effect on *I*_*K*,*ACh*_ at hyperpolarized versus depolarized membrane voltages. This led to a slowing of the pacemaker rate due to an attenuated slope of phase 4 depolarization with only marginal effect on action potential duration and amplitude. We also simulated the theoretical effects of genetic variants that alter the voltage sensitivity of M2R. Modest negative shifts in voltage sensitivity, predicted to increase the affinity of the receptor for ACh, slowed the rate of phase 4 depolarization and slowed heart rate, while modest positive shifts increased heart rate. These simulations support our hypothesis that altered M2R voltage sensitivity contributes to disease and provide a novel mechanistic foundation to study clinical disorders such as atrial fibrillation and inappropriate sinus tachycardia.

## Author summary

Heart rate regulation is dependent upon a delicate interplay between parasympathetic and sympathetic nerve activity at the level of the sinus node. Acetylcholine slows the heart rate by activating the M2 muscarinic receptor (M2R) that, in turn, opens the acetylcholine-activated potassium channel (*I*_*K*,*ACh*_) to slow the firing of the sinus node. Surprisingly, the M2R is sensitive to membrane potential and undergoes conformational changes throughout the cardiac action potential that alter the affinity for acetylcholine, with secondary consequences for *I*_*K*,*ACh*_ activity. Here, we investigated the contribution of M2R voltage sensitivity to the rate and shape of the human sinus node action potential in physiological and pathophysiological conditions, using a Markovian model of the *I*_*K*,*ACh*_ channel integrated into a computational model of human sinus node. The computational model allowed us to assess the effects of potential genetic variants that alter specific properties of voltage sensitivity. Our results indicate that alterations in the M2R voltage sensitivity play a significant role in the physiology and pathophysiology of the human sinus node and atria. Our computational model is relevant for future studies aimed at the design and development of anti-arrhythmic agents that specifically target the unique voltage-sensitive properties of M2R.

## Introduction

The cardiac muscarinic receptor (M2R) plays a crucial role in regulating heart rate variability and vulnerability to atrial arrhythmia by modulating the acetylcholine (ACh) activated K^+^ current *I*_*K*,*ACh*_. Cardiac K_ACh_ channels are heteromultimers composed of two G-protein-coupled inward rectifier K^+^ channel subunits, Kir 3.1 and Kir 3.4 [1]. ACh activation of M2R triggers dissociation of the G beta-gamma subunits (G_βɣ_) that in turn directly activate Kir 3.x subunits to conduct *I*_*K*,*ACh*_. Unexpectedly, M2Rs were discovered to possess an intrinsic ability to sense transmembrane voltage [2] and the affinity of the receptor for ligands was noted to vary in response to changes in membrane voltage [3]. In particular, the affinity of the receptor for ACh is increased at hyperpolarized membrane potentials and decreased at depolarized potentials. The changes in affinity exert a downstream effect on the K_ACh_ channel such that the channel is more active (more current) at hyperpolarized potentials and less active (less current) at depolarized potentials. The observation that M2Rs are intrinsically voltage sensitive has profound implications for cellular signaling in excitable tissues, such as heart. For example, voltage sensitive behavior provides a mechanistic explanation for a decades-old enigmatic process called *I*_*K*,*ACh*_ “relaxation” gating. Relaxation gating refers to a time-dependent change in current magnitude following a depolarizing or hyperpolarizing voltage step [4] and has important consequences for shaping the cardiac action potentials (AP), especially in the sinus node. We recently proposed that relaxation gating represents a voltage dependent change in ACh affinity induced by voltage dependent conformational changes within M2R [5]. Our experimental data provide a mechanistic basis to explain the participation of *I*_K,ACh_ in the modest chronotropic effects induced by resting vagal tone. As a result of conformational changes in the M2R, the affinity for ACh varies throughout the cardiac electrical cycle such that low (subsaturating) ACh concentrations preferentially activate *I*_K,ACh_ during diastolic membrane voltages thereby slowing the spontaneous firing rate without appreciably altering AP duration (APD).

Alterations in the voltage sensitivity of M2R could theoretically contribute to cardiovascular diseases that clinically present with apparent changes in vagal tone. For example, genetic variants in M2R that shift the receptor occupancy into the hyperpolarized state would be expected to increase the affinity of the receptor for ACh and thus activate more K_ACh_ channels at a given ACh concentration (or degree of vagal tone). Accordingly, genetic variants in M2R that shift the receptor occupancy into the hyperpolarized state might explain the clinical phenotype of vagally-mediated atrial fibrillation (AF), patients who present with bradycardia in the setting of physiological (basal) ACh concentrations. Alternatively, genetic variants in M2R that shift the receptor occupancy into the depolarized state would be expected to decrease the affinity of the receptor for ACh and thus fewer K_ACh_ channels activate at a given ACh concentration (or given degree of vagal tone). This would decrease the effects of vagal modulation of heart rate, thereby increasing basal heart rate, as observed in the syndrome of inappropriate sinus tachycardia (IST).

To provide insights into the contribution of M2R voltage sensitivity to cardiac electrophysiology in physiological and pathophysiological conditions, we extended our previous Markovian model of M2R [5] to incorporate G_βɣ_-mediated activation of the K_ACh_ channel and integrated the revised Markovian model into a human model of the sinus node (SN) cell model [6]. Based on experimental data from isolated human SN cells [7, 8], Fabbri and colleagues recently published a comprehensive model of the human SN pacemaker cell that faithfully recapitulated the effects of autonomic modulation as well as mutations associated with SN dysfunction [6]. In the Fabbri model and its parent model [9], *I*_*K*,*ACh*_ is described by a voltage- and [ACh]-dependent gate, but the intrinsic voltage sensitivity of M2R is not incorporated. Here, we introduce a model reproducing the effects of M2R voltage sensitivity on human SN cell function under physiological and pathophysiological conditions. These simulations support our hypothesis that altered M2R voltage sensitivity contributes to disease and provide a novel mechanistic foundation to study clinical disorders such as AF and IST.

## Results

### Effects of low concentrations of ACh on sinus node action potentials

**Fig 1:**
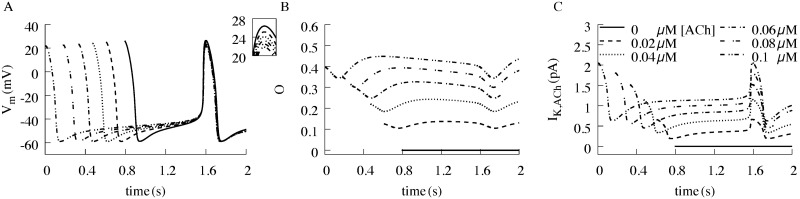
Simulation of the effect of varying amounts of ACh concentrations on sinus node properties. (A) Resulting transmembrane voltage (V_m_) showing the increase in BCL from 827 ms to 1585 ms with a reduction of APD_90_ by roughly 10%. (B) Open probability O of the channel and (C) the corresponding activated *I*_*K*,*ACh*_.

For decades, the contribution of *I*_*K*,*ACh*_ to the modest chronotropic effects of ‘physiological’ or low-dose ACh has been debated [10–12]. Based on the M2R voltage-dependent properties, we predict that subsaturating ACh concentrations exert a larger effect during diastolic (hyperpolarized) membrane voltages, compared to the voltages during the cardiac AP (depolarized) [3, 5]; thus preferentially slowing the pacemaker rate with minimal effect on APD. To test this hypothesis, we simulated the effects of varying low, sub-saturating concentrations (e.g., 20–100 nM) of ACh on sinus node AP properties (Fig 1). The most striking effect of increasing ACh concentration was reduced slope of phase 4 depolarization and the corresponding increase in basic cycle length (Fig 1A, Table 1, V_*shift*_ = 0), with minimal shortening of APD_90_ (Table 2, V_*shift*_ = 0). Thus, the basic cycle length (BCL) increased from 827 ms in the absence of ACh, to 1585 ms in the presence of 0.1 μM ACh. The AP amplitude decreased steadily by up to 4.7 mV at the highest tested concentration of 0.1 μM ACh (inset of Fig 1A). Simulated open probability *O* and *I*_*K*,*ACh*_ for different ACh concentrations are shown in Fig 1B and 1C. Taken together, these simulations indicate that subsaturating concentrations of ACh slow spontaneous excitation of the SN cell by inhibiting the rise of phase 4 depolarization, without appreciably shortening APD_90_ or reducing the amplitude of the AP.

**Table 1:** Basic cycle lengths (ms) at different concentrations of ACh (μM) and shifts in the voltage affinity of M2R (mV).

*V*_*shift*_ [ACh]	-150	-30	-10	0	+10	+30	+150
0	827	827	827	827	827	827	827
0.02	1122	1063	1017	994	973	944	921
0.04	1414	1279	1177	1126	1081	1019	968
0.06	1825	1530	1348	1262	1189	1090	1009
0.08	3307	1882	1545	1411	1304	1162	1048
0.1	-	2705	1802	1585	1428	1235	1085

**Table 2:** APD_90_ (ms) at different concentrations of ACh (μM) and shifts in the voltage affinity of M2R (mV).

*V*_*shift*_ [ACh]	-150	-30	-10	0	+10	+30	+150
0	150	150	150	150	150	150	150
0.02	146	145	146	145	146	146	147
0.04	138	141	141	142	143	144	144
0.06	136	137	139	139	140	140	142
0.08	132	134	135	138	137	138	139
0.1	-	130	134	134	135	137	138

### Modeling perturbations in M2R voltage sensitivity

**Fig 2:**
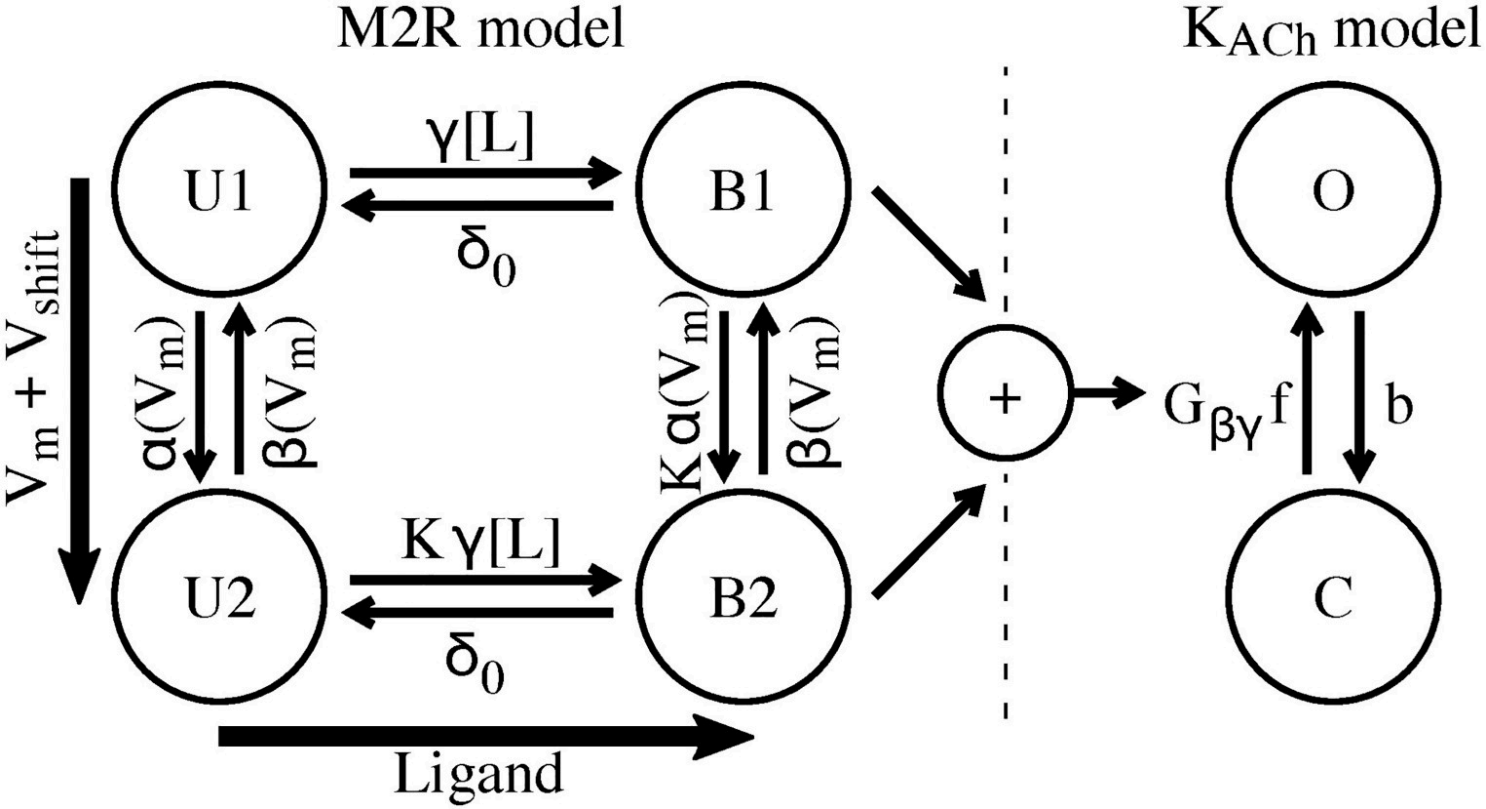
Markovian model describing the response of K_ACh_ channels to different ACh concentrations and membrane voltages. The left sub-model describes the M2R, with states U1 and B1 for high ACh affinity and hyperpolarized voltages, and states U2 and B2 for low ACh affinity and depolarized voltages. The right sub-model represents the K_ACh_ channel with the open state O and the closed state C. For further details, see the method section.

**Fig 3:**
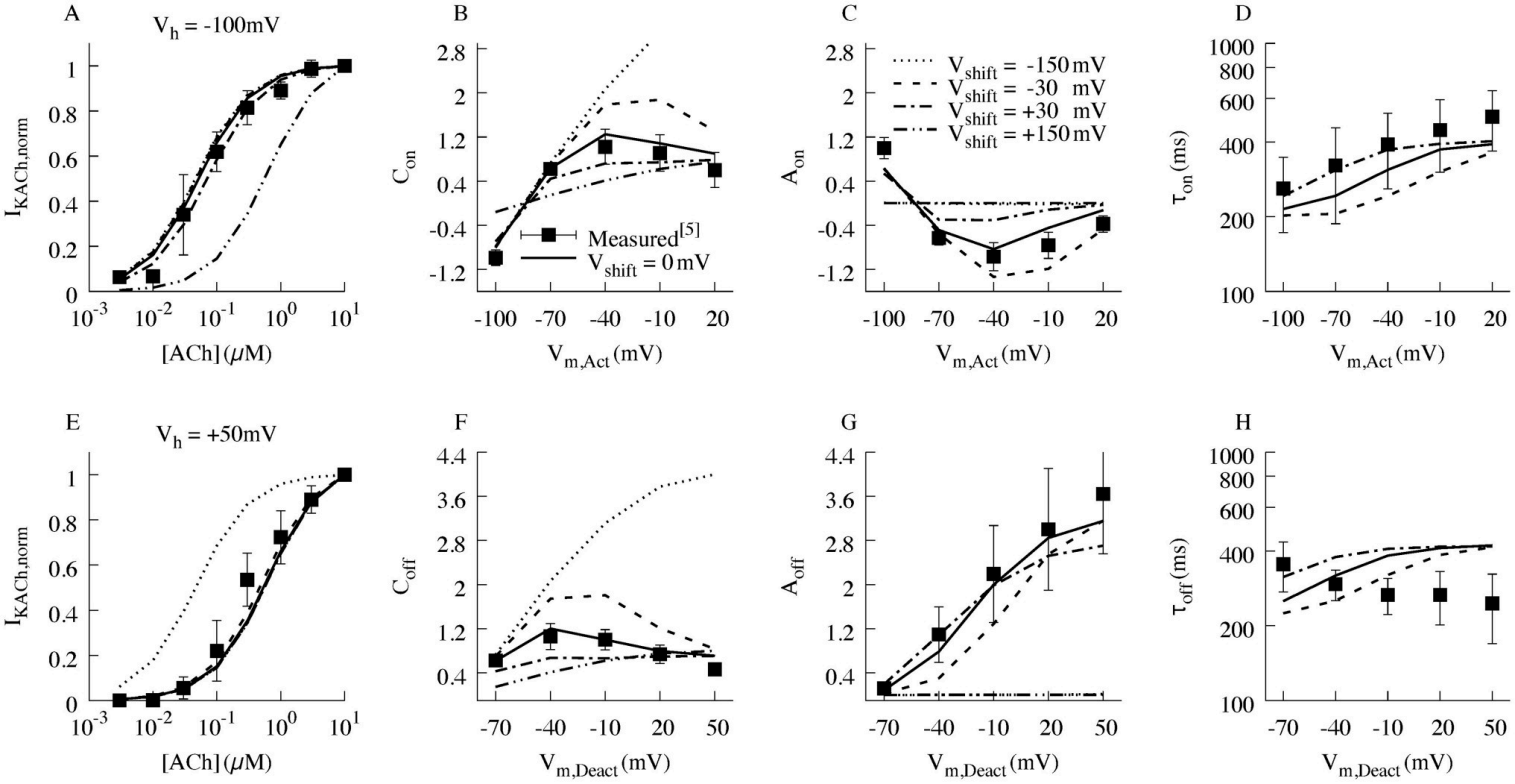
Concentration-response curves and relaxation gating features. Concentration-response curves at a holding potential of (A) -100 mV and (E) +50 mV. Relaxation gating features derived from the mono-exponential fitting of activation and deactivation traces (B,C,F,G). The associated relaxation kinetic time constants are shown in (D,H). Measured data is represented as mean ± std. Respectively the squared fit error for each feature is; C_on_: 0.06, C_off_: 0.025, A_on_: 0.11, Aoff: 0.014, τ_on_: 0.04, τ_off_: 0.18, I_KACh,norm,-100mV_: 0.003, I_KACh,norm,50mV_: 0.03. The total error of the features is equal to the square root of the sum of the respective squared fit errors (Eq 11 and Methods).

In our previous experimental studies using isolated feline left atrial myocytes, the voltage dependence of M2R was explored by measuring the ACh concentration-*I*_*K*,*ACh*_ response relationship at hyperpolarized (-100 mV) and depolarized membrane voltages (+50 mV) [3, 5]. These experiments indicated that the affinity of the receptor for ACh was greater at hyperpolarized membrane voltages, compared to depolarized voltages. We reasoned that, similar to voltage-gated ion channels, putative disease-associated mutations in M2R might alter the voltage sensitivity of the M2R, with unique consequences for sinus node AP properties and heart rate responses. We modified rate parameters (Eqs 5 and 6) to shift the receptor occupancy towards a hyperpolarized state (higher affinity) or a depolarized state (lower affinity) (S1A Fig). Thus, we simulated the effects of shifting the M2R voltage sensitivity (Fig 2, S3C Fig). S1B Fig highlights the effects of voltage shifts on the state O. Negative shifts (e.g., *V*_*shift*_ of -30 mV and -150 mV), which caused a more hyperpolarized state of the receptor, increased the occupancy of the U1 and B1 states, as well as the state O. Negative voltage shifts resulted in a slight leftward shift in the concentration-response curve when the cell was held at +50 mV (Fig 3E). Likewise, positive shifts in M2R voltage sensitive parameters caused a slight rightward shift in the concentration-response curve for a holding potential of V_h_ = -100 mV (Fig 3A). To avoid local minima during the parameter fitting, the model was forced to favor the open state (state *O* equal to 1), at the maximum concentration of 10 μM ACh and to favor the closed state (*O* equal to 0), with no ACh present. Further, U1 was forced to be as high as possible at a holding potential of -100mV and U2 at +50 mV, in the absence of ACh. Thus, negative and positive voltage shifts did not move the steady-state concentration-response relationships outside of these ranges. Notwithstanding, the voltages experienced by the single cell model vary between -60 and +30 mV (Fig 1A), within the minimum and maximum ranges defined by the parameter fitting.

We previously described the kinetics of “relaxation” gating of *I*_*K*,*ACh*_ in terms of activation and deactivation of K_ACh_ channels in the setting of subsaturating ACh concentrations [5]. Activation kinetics were measured by first stepping to a depolarized voltage (+60 mV) to close a large portion of K_ACh_ channels at a physiological voltage, followed by stepping through a range of voltages to measure activation of *I*_*K*,*ACh*_. Deactivation kinetics were assessed by a pre-pulse to a hyperpolarized voltage (-100 mV) to open channels, followed by variable test voltage steps to measure the rate of K_ACh_ channel closure. Accordingly, simulated *I*_*K*,*ACh*_ evoked by 0.1 μM ACh using the activation and deactivation voltage protocols are presented in S1 Fig and the kinetic parameters are described in Table 3. The simulations recapitulate the experimental features of *I*_*K*,*ACh*_ relaxation gating [5], as shown in S1 Fig. The effects of voltage shifts in M2R voltage sensitive parameters on *I*_*K*,*ACh*_ relaxation gating parameters are shown in Fig 3.

**Table 3:** Model parameters resulting from stochastic optimization.

Parameter	Value	Initial Value
*α*_0_	230376 [1/s]	10000 [1/s]
*β*_0_	11275 [1/s]	10000 [1/s]
*z*_*α*_	0.808	0.1
*z*_*β*_	0.508	0.1
*γ*_0_	10 [1/(s μM)]	10 [1/(s μM)]
*δ*_0_	11.64 [1/s]	5 [1/s]
K	0.0727	0.1
f	79.92 [1/s]	100 [1/s]
b	3.3 [1/s]	1 [1/s]

**Fig 4:**
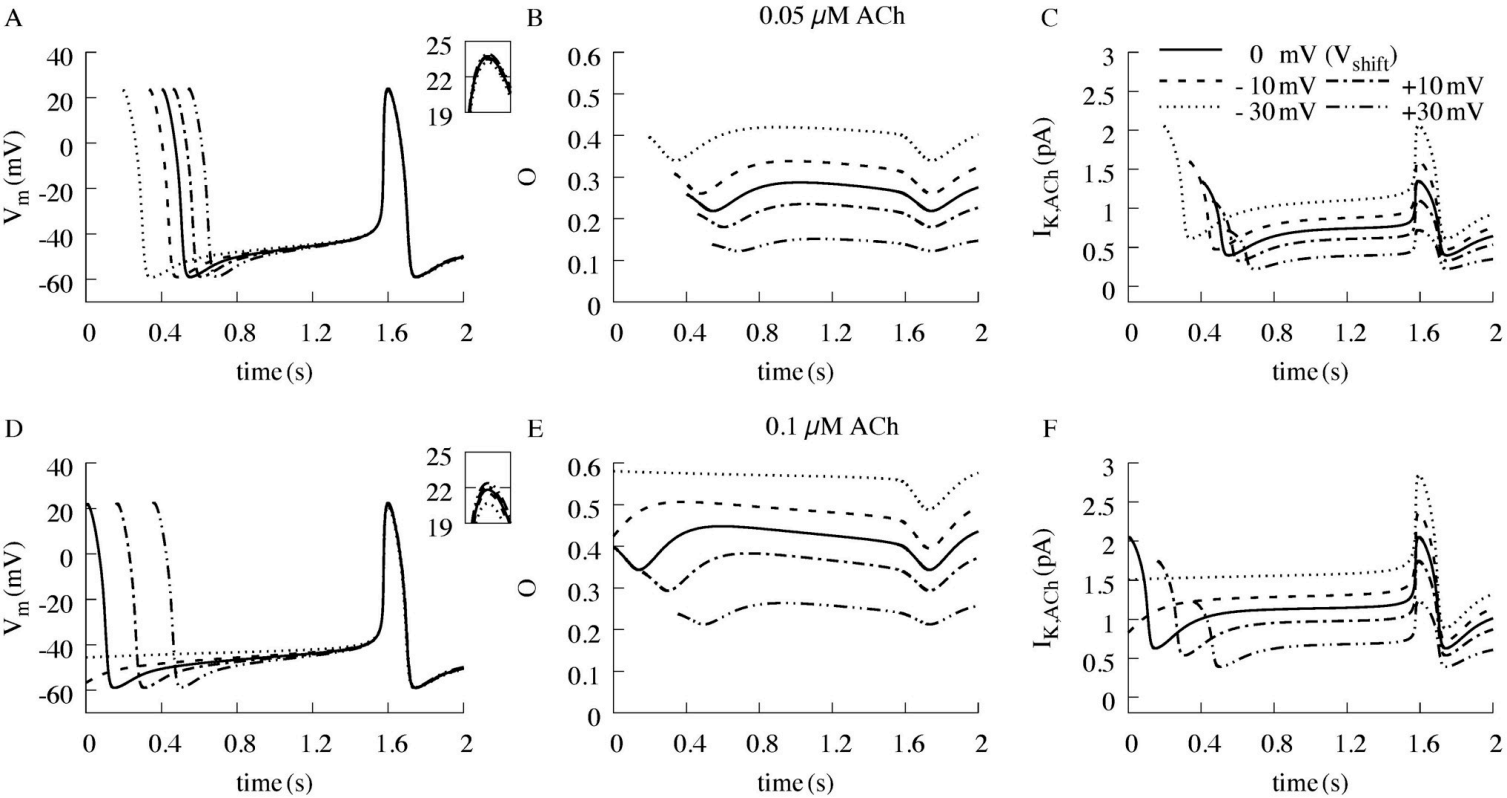
Effect of positive and negative voltage shifts of the M2R on the I_*K*,*ACh*_. In the presence of (A,B,C) 0.05 μM ACh and (D,E,F) 0.1 μM ACh. (A,D) present the resulting sinus node APs, (B,E) the open probability O of the K_ACh_ channel and (C,F) the resulting *I*_*K*,*ACh*_.

Next, we simulated the effects of negative and positive voltage shifts in M2R voltage-sensitive parameters on sinus node APs, together with the corresponding effects on state *O* and *I*_*K*,*ACh*_ (Fig 4). Negative shifts (e.g., V_*shift*_ of -10 mV and -30 mV), which would be predicted to increase ACh affinity, reduced the slope of phase 4 depolarization in a concentration-dependent manner, with minimal effects on AP amplitude or APD_90_ (Fig 4A versus 4D, Tables 1 and 2). The reduction in the slope of phase 4 depolarization induced by a negative V_*shift*_ was due to an increase in the open probability of K_ACh_ channels relative to the control condition, thereby increasing the magnitude of *I*_*K*,*ACh*_. These results indicate that shifts in the M2R voltage sensitivity impact the rate of spontaneous depolarization of sinus node APs.

**Fig 5:**
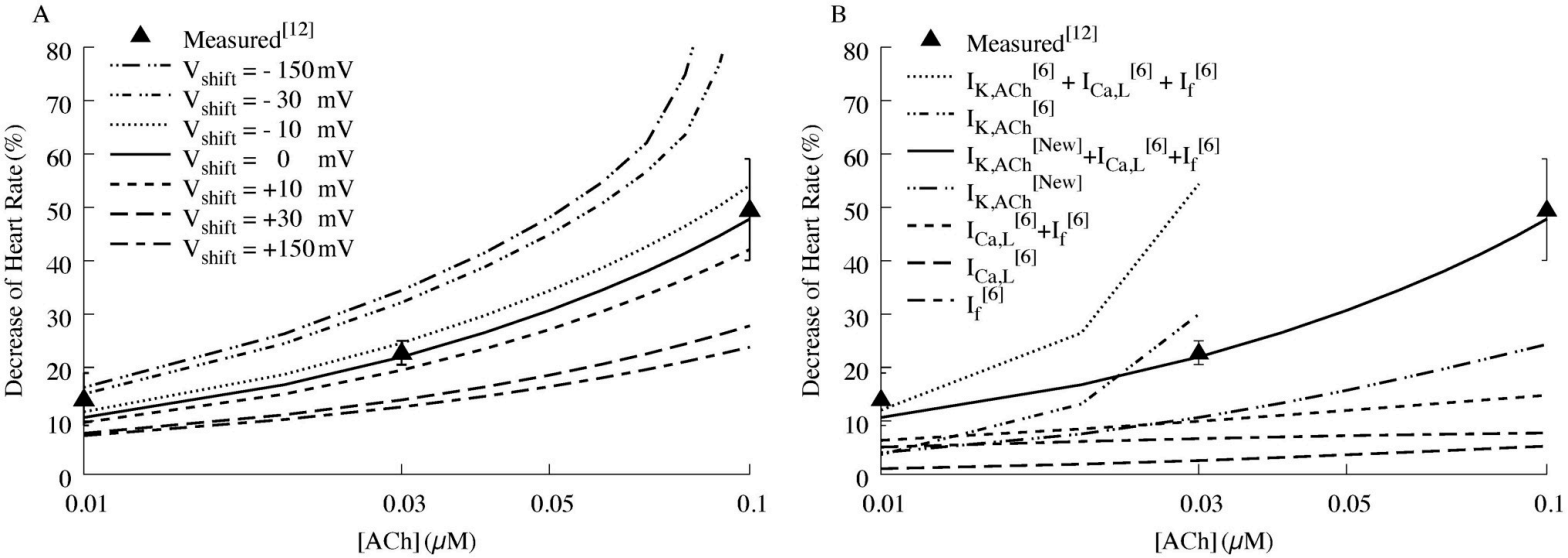
Comparison of simulated to measured ACh concentration-heart rate response in humans. (A) Concentration-heart rate response to the effect of variable M2R voltage sensitivity. Negative voltage shifts accentuate the response of ACh, while positive shifts blunt the heart rate slowing induced by ACh. (B), The individual contributions of ACh-sensitive currents to the measured concentration-heart rate response. Our current model that incorporates the voltage-sensitivity of the M2R into I_*K*,ACh_ (new), together with formulations for the ACh-sensitive currents I_Ca,L_ and I_f_ from Fabbri et al [6] provides the best fit to the measured data. I_*K*,ACh_ contributes to about 50% of the heart rate response at 0.03 μM ACh and contributes a greater proportion to heart rate slowing at higher ACh concentrations.

To further characterize the physiological consequences of positive and negative voltage shifts, we studied the effects of variable M2R voltage sensitivity on the ACh concentration-heart rate response relationship (Fig 5A). Our model recapitulates experimental data [12] indicating that ACh concentrations ranging from 0.01 to 0.1 μM induce slowing of the spontaneous activity by 10% and 45%, respectively. Hyperpolarizing shifts in the M2R voltage dependent parameters shifted the relationship toward progressive spontaneous activity slowing. By contrast, depolarizing shifts in these parameters antagonized the spontaneous activity slowing induced by ACh. Taken together, these results suggest that shifts in M2R voltage sensitive parameters exert significant physiological effects on SN firing rate and AP parameters.

Next, we quantified the effects of the individual ACh-influenced cardiac currents on heart rate slowing. Fig 5B illustrates the relative contributions of ACh-sensitive currents, including our new model of I_K,ACh_, together with formulations of the L-type Ca2+ current (I_Ca,L_) and the hyperpolarized-activated ‘funny current’ (I_f_) from Fabbri et al. [6]. Inspection of the individual contributions of the ACh-sensitive currents reveals that our model of I_K,ACh_ (incorporating the voltage-sensitivity of M2R) accounts for roughly 50% of the slowing at ~30nM and more for higher concentrations. These results highlight the important contribution of M2R voltage sensitivity to heart rate slowing induced by ACh.

## Discussion

The observation that M2Rs are intrinsically voltage sensitive has profound implications for cellular signaling in excitable tissues, such as heart. This could have important consequences for cardiovascular drug development in that the affinity for ligands may vary in a voltage- and ligand-specific manner. The properties of M2R voltage dependence provide a novel molecular basis to explain the previously perplexing “relaxation” gating of *I*_*K*,*ACh*_, originally described in the 1970s [4]. Precisely how the receptor’s voltage dependent properties influence the firing rate and shape of sinus node APs under physiological conditions, or in the presence of a M2R genetic defect, remains unknown. In order to address this question, we enhanced our previous Markovian model of M2R [5] by a 2-state Markovian model to incorporate G-protein activation of the K_ACh_ channel. The parameters of this revised model were adapted to experimental data from isolated feline left atrial myocytes, see Methods. We integrated the revised model into a human SN single cell model and used computational simulations to gain insights into effects of voltage sensitivity of M2Rs.

While there is no controversy as to the cardiac effects of strong vagal stimulation, the participation of *I*_*K*,*ACh*_ in mediating the purely chronotropic effects of low (or “physiologic”) ACh concentrations has been debated over the past thirty years. Low concentrations of ACh (e.g., below 100 nM) and weak vagal stimulation reduce spontaneous pacemaker rate without altering APD or increasing the maximal diastolic potential [10]. DiFrancesco et al. proposed that weak vagal stimulation primarily inhibited *I*_*f*_ with little to no contribution from *I*_*K*,*ACh*_. Indeed, the *EC*_*50*_ for ACh modulation of *I*_f_ is an order of magnitude lower than that required for *I*_*K*,*ACh*_ activation [11]. However, subsequent studies have corroborated an important role for *I*_*K*,*ACh*_ in mediating the chronotropic effects of weak vagal stimulation and externally applied low ACh concentrations [12]. Perhaps the strongest evidence for *I*_*K*,*ACh*_ contribution to basal chronotropy comes from the Kir3.4 knock-out mouse which displays a specific deficit in heart rate variability at rest [13, 14]. These studies confirm that low ACh concentrations (basal vagal tone) activate *I*_*K*,*ACh*_ to slow heart rate. Our experimental and simulated data provide a mechanistic basis to explain the participation of *I*_*K*,*ACh*_ in the modest chronotropic effects induced by resting vagal tone. Our experimental data predict that the affinity of M2R for ACh varies throughout the AP. The simulations performed here corroborate that subsaturating ACh concentrations preferentially open the K_ACh_ channel during diastolic membrane voltages and thereby slow the spontaneous firing rate. As seen in Fig 5B, I_K,ACh_ accounts for roughly half of the heart rate slowing at concentrations around 30 nM, and more for higher concentrations.

*In silico* modeling is valuable for understanding fundamental features of physiology and pathophysiology [15–17]. This is especially relevant for modeling of *I*_*K*,*ACh*_ in disease states. For example, numerous disease-causing mutations in ion channel genes alter the voltage sensitivity of the channel [18, 19]. In light of these observations, we simulated effects of hypothetical mutation-induced positive and negative shifts in M2R voltage sensitivity to ACh on sinus node AP properties and spontaneous firing. Modest voltage shifts (*V*_*shift*_ of ±10 mV) exerted significant effects on the slope of phase 4 depolarization and thus on spontaneous activity responses to subsaturating ACh concentrations, such as those elicited by vagal resting tone. Moderate voltage shifts (*V*_*shift*_ of ± 30 mV) caused more profound changes in spontaneous activity. These simulations provide a proof-of-principle for a theoretical contribution of altered M2R voltage sensitivity to cardiovascular disease states associated with changes in vagal tone. For example, parasympathetic induction and maintenance of atrial arrhythmias is a well-described phenomenon, first reported in 1930 [20]. Moreover, increased parasympathetic tone is an initiating factor in a subset of AF patients [21]. Our simulations suggest that genetic variants in M2R that shift the receptor occupancy towards hyperpolarized states (U1/B1) associated with increased ACh affinity of the receptor increase I_KACh_ at a given ACh concentration (or degree of vagal tone). These genetic variants might explain, in part, the clinical phenotype of vagally-mediated AF patients who present with bradycardia in the setting of physiological (basal) ACh concentrations. Also, our simulations suggest that genetic variants in M2R that shift receptor occupancy towards depolarized states (U2/B2) associated with decreased ACh affinity of the receptor reduce I_KACh_ at a given ACh concentration (or degree of vagal tone). Thus, positive shifts in M2R voltage sensitivity would decrease the effects of vagal modulation of heart rate, thereby increasing basal heart rate, as observed in the syndrome of IST. Indeed, a recent study confirmed decreased parasympathetic tone in IST patients [22]. While speculative, the hypothesis that altered M2R voltage sensitivity is relevant for cardiac diseases provides a novel mechanistic foundation to study disorders such as AF and IST.

### Limitations of the model

There are several limitations inherent in the application of the model to describe the kinetics and behavior of *I*_*K*,*ACh*_. First, the receptor-channel model was fit and optimized to recreate the kinetics of *I*_*K*,*ACh*_ occurring at a concentration of 0.1 μM ACh. While this is within the range of measured concentrations of ACh, measurements at lower concentrations could enable a more accurate reconstruction of the behavior of *I*_*K*,*ACh*_ and a direct comparison to the effect of *I*_*f*_ at such concentrations. Furthermore, we acknowledge that the description of the process of dissociation of G_βɣ_ from the M2R to the activation of the K_ACh_ channel is highly simplified. We used a simple channel opening description with a 2-state Markovian model neglecting different binding properties of different Kir subunits or cooperativity mechanisms in binding of G_βɣ_. This simplification was necessary as parameters for more complex models are not identifiable with the existent experimental data. Also, the model is not able to reproduce the distinct characteristics of the deactivation protocol time constants. The experimental data indicate that the deactivation time constant is nearly voltage-independent (Fig 3H), the model predicts an increase with membrane depolarization. Nonetheless, because the other simulated features have a very high similarity to the measured values (Fig 3B, 3C, 3F and 3G) and the time constants are in the range of less than half the length of an action potential, we believe that any error introduced does not significantly influence our findings. Additionally, dissociation or binding of G_βɣ_ in our model does not account for changes in the process due to other influences or any binding to sites other than the K_ACh_ channel.

The SN cell model recapitulates the experimental findings that ACh inhibits *I*_*f*_ and *I*_*Ca*,*L*_, which also contribute to slowing of spontaneous pacing rate [23]. We argue that unlike *I*_*K*,*ACh*_, the M2R voltage dependent effects do not influence *I*_*f*_ and *I*_*Ca*,*L*_ on the time scale of the AP. *I*_*f*_ and *I*_*Ca*,*L*_ inhibition are mediated by inhibition of the cAMP-dependent protein kinase A cascade that functions on a much slower time scale than the APD. By contrast, our simulations demonstrate that voltage dependent conformational changes in M2R influence *I*_*K*,*ACh*_ throughout the cardiac AP, modulating both firing rate and APD. Finally, because our model does not fully integrate all the components of the autonomic nervous system, it is possible that the effects of the putative genetic mutations might be mitigated by compensatory changes in the autonomous nervous system’s response to changes in heart rate.

### Conclusions

The recent observation that M2Rs are intrinsically voltage sensitive has important implications for understanding the physiology and pathophysiology of parasympathetic regulation of heart rate and APD. By optimizing and integrating a new Markovian model into a human SN model, we show that low ACh concentrations preferentially slow beating rate, without shortening APD, and thereby provide additional support that *I*_*K*,*ACh*_ participates in the purely chronotropic effects of basal vagal tone. Moreover, we explore the effects of altered M2R voltage sensitivity and provide a proof-of-principle foundation that altered sensitivity could result in clinical manifestations of disease states such as vagally-mediated atrial fibrillation and syndrome of inappropriate sinus tachycardia. Given the importance of parasympathetic regulation of atrial vulnerability, M2Rs represent an important therapeutic target to control or prevent atrial arrhythmias.

## Methods

### Model of M2R and KACh channels

We developed a Markovian model to reconstruct the behavior of K_ACh_ channels at different ACh concentrations and varying transmembrane voltages (Fig 2). The model comprises 3 sub-models: (1) A Markovian model describing the kinetics of the M2R depending on different concentrations of ACh at different voltages (M2R model), (2) a Markovian model describing the activation of the K_ACh_ channel based on dispersion of G_βɣ_ protein from the receptor to the channel (K_ACh_ channel model), and (3) a model of potassium current through K_ACh_ channels.

#### Markovian model of M2R

This sub-model describes biophysical properties of the M2R and comprises 4 distinct states, *U1*, *B1*, *U2* and *B2*, whose interaction describes the affinity of M2R to changes in ACh concentration *[L]* and transmembrane voltage *V*_*m*_ (Fig 2, left side). The sub-model was published previously [5] and is a simplification of a more complex model of receptor systems [24]. Time-dependent changes of states were defined as:

$$\frac{dU1}{dt}=-(\alpha (V_{m})+\gamma_{0}\;[L])\;U1+\delta_{0}\;B1+\beta (V_{m})\;U2$$ (1)

$$\frac{dB1}{dt}=-(K\;\alpha (V_{m})+\delta_{0})\;B1+\gamma_{0}\;[L]\;U1+\beta (V_{m})\;B2$$ (2)

$$\frac{dU2}{dt}=-(\beta (V_{m})+K\;\gamma_{0}\;[L])\;U2+\alpha \;{(V}_{m})U1+\delta_{0}\;B2$$ (3)

$$\frac{dB2}{dt}=-(\beta (V_{m})+\delta_{0})\;B2+K\;\alpha (V_{m})\;B1+K\;\gamma_{0}\;[L]\;U2$$ (4)

with the transition rate coefficients *α(V*_*m*_*)*, *β(V*_*m*_*)*, *and γ*_*0*_*[L]*, and the constant parameters δ_0_ and *K*. Each of the 4 distinct states represents the M2R under specific conditions. The state *U1* represents the M2R at hyperpolarized *V*_*m*_ without bound ACh, *U2* the M2R at a depolarized *V*_*m*_ without bound ACh, *B1* the M2R at a hyperpolarized *V*_*m*_ with bound ACh, and *B2* the M2R at depolarized *V*_*m*_ with bound ACh.

The transition rate coefficients between these states are dependent on *V*_*m*_ or *[L]*. The transition rate coefficients *α* and *β* between *U1* and *U2 are* dependent on *V*_*m*_:

$$\alpha (V_{m},V_{shift})=\alpha_{0}\;exp\left(z_{\alpha }\;\frac{(V_{m}+V_{shift})F}{RT}\right)$$ (5)

$$\beta (V_{m},V_{shift})=\beta_{0}\;exp\left({-z}_{\beta }\;\frac{(V_{m}+V_{shift})F}{RT}\right)$$ (6)

with the rates *α*_*0*_ and *β*_*0*_, the charges *z*_*α*_ and *z*_*β*_, the Faraday constant *F*, gas constant *R* and temperature *T*, see S3B and S3C Fig for the respective traces. Additionally to the original formulation, we introduced a voltage shift *V*_*shift*_ to describe effects of mutations on voltage sensitivity of the receptor. The shift solely affects the voltage dependent transition rate coefficients of the M2R and thus indirectly influences the states of the K_ACh_ channel model and the resulting *I*_*K*,*ACh*_. We defined negative *V*_*shift*_ as hyperpolarizing shifts. Negative *V*_*shift*_ lead to an increased occupancy of the states U1 and B1, i.e. to a more hyperpolarized state of the receptor model. Likewise, positive *V*_*shift*_ were defined as depolarizing shifts as they lead to increased occupancy of the states U2 and B2, i.e. to a more depolarized state of the receptor model.

A total removal of voltage sensitivity was achieved for large shifts locking the M2R in its hyperpolarized state (*V*_*shift*_ ≦ -150 mV) and its depolarized state (*V*_*shift*_ ≧ +150 mV) (S3A Fig).

A change in voltage affinity due to the presence of ACh was represented as a change in the transition rate. The transition rate coefficient *α* was multiplied with the constant *K* in the voltage dependent transition between B1 and B2. Similarly, the ACh affinity of the M2R was represented by the transition rate coefficients *δ*_0_ and *γ*_0_.

#### Markovian model of K_ACh_ channels

Simulating physiological mechanisms, the K_ACh_ channel model was coupled to the M2R model via a description of G_βɣ_ protein. The normalized concentration of G_βɣ_ was represented as the sum of the states *B1* and *B2* of the M2R model. The K_ACh_ channel model comprised an open (O) and closed (C) state (Fig 2, right side). The model described the channel kinetics based on the dispersion of G_βɣ_ causing opening of the K_ACh_ channel:

$$\frac{dO}{dt}=f\;(B1+B2)\;(1-O)-b\;O$$ (7)

with forward rate *f* and the backward rate *b*.

#### Modeling of *I*_*K*,*ACh*_

A previously developed description for *I*_*K*,*ACh*_ was used to calculate this current [23]. The description of *I*_*K*,*ACh*_ includes its dependency on extracellular K^+^ concentration *[K*^+^*]*_*o*_ and rectification:

$$I_{K,ACh}=g_{K,ACh}\;(\frac{{{[K}^{+}]}_{o}}{10\;mM+{{[K}^{+}]}_{o}})\;\frac{V_{m}-E_{K}}{1+exp\left[{(V}_{m}-E_{K}-140\;mV\right)\;F/2.5RT]}$$ (8)

with the Nernst potential *E*_*K*_. We calculated the conductance of K_ACh_ channels as the maximum conductance *g*_*K*,*ACh*,*max*_ multiplied with the open probability *O* from the K_ACh_ channel sub-model:

$$g_{K,ACh}=g_{K,ACh,max}\;O$$ (9)

### Measured data and model parameterization

The parameters of the model were determined by iterative stochastic optimization as previously described [5]. Model parameterization was implemented in Matlab R2017a (The Mathworks Inc., Natick, MA) and the Matlab Parallel Computing Toolbox. An error function based on the root mean squared differences of the measured versus simulated features of the activation protocol, deactivation protocol, and concentration response curve from [5] was minimized.

Measured features were based on whole-cell voltage-clamp experiments [5]. *I*_*K*,*ACh*_ for the activation and deactivation clamp protocols was recorded in the presence of 0.1 μM ACh. The 3 best measured currents, see S1 Fig, from [5] of each protocol were then fitted to a mono-exponential equation, averaged and then normalized to C_off_ at -10 mV:

$$I(t)=C+A\;exp\left(-\frac{t}{\tau }\right)$$ (10)

with the constants *A* and *C*, and the time-constant τ of activation (on) and deactivation (off). We choose this strategy, as extracting mono-exponential features and then averaging them introduces less error in the overall reconstructed behavior than averaging the signals themselves. The same fitting approach was used during the model parameterization.

Concentration response curves were measured as the resulting *I*_*K*,*ACh*_ at either a holding potential of -100 mV or +50 mV at different ACh concentrations (Fig 5A and 5E). The currents were then normalized to the current measured at maximal ACh concentration for each holding potential.

A total of 8 simulated features f_s,i_, consisting of six features for the voltage clamp protocols (i.e. C_on_, C_off_, A_on_, A_off_, τ_on_, and τ_off_) and two for the concentration response curves (i.e. I_KACh,norm,50mV_ and I_KACh,norm,-100mV_), were compared to their corresponding measured features f_m,i_ (Fig 5B–5D and 5F–5H):

$$\begin{array}{ll} E^{2}= & \sum_{i=1}^{8}{\left(\frac{||f_{m,i}-f_{s,i}|{|}_{2}}{||f_{s,i}|{|}_{2}}\right)}^{2}\\ & +\left(1-max\left(O\right)\right)+{\left({\left(1-max_{50mV}\left(U2\right)\right)}^{2}+{\left(1-max_{-100mV}\left(U1\right)\right)}^{2}\right)}_{0\mu M}\\ & +({\left(1-max_{50mV}\left(B2\right)\right)}^{2}+{\left(1-max_{-100mV}\left(B1\right)\right)}^{2}\\ & +\left(1-{\left(B1+B2\right)}^{2}\right){)}_{10\mu M} \end{array}$$ (11)

Other components of the cost function were used to constrain the behavior of the model at specific ACh concentration and *V*_*m*_, and to ensure high open probability of the channel. Without bound ACh and *V*_*m*_ of +50 mV, the state U2 was forced to be maximal. Respectively, *U1* was forced to be maximal without bound ACh and a transmembrane voltage of -100 mV. Equivalently, with bound ACh, state *B2* and *B1* were forced to be maximal at -100 mV and 50 mV, respectively. Further, the sum of *B1* and *B2* was forced to be maximal with bound ACh.

### Single cell simulations

For simulations of single cell electrophysiology, the new receptor-channel model was integrated in the publically available model of human SN cells [6]. The formulations for the effect of ACh on *I*_*CaL*_ and *I*_*f*_ were left unaltered throughout the experiments. The model was first exported to Matlab using OpenCOR (www.opencor.ws) and then modified accordingly. Numerical integration was performed by using the integrated ode15s formulation provided by Matlab. We measured BCL to characterize the rate of spontaneous activation of the simulated SN cell at varying concentration of ACh and *V*_*shift*_. The maximum conductivity *g*_*K*,*ACh*,*max*_ was set 0.0022 μS to reproduce previously published heart rate slowing in the presence of 0−0.1 μM ACh [12]. Furthermore, we measured the APD at 90% repolarization (APD_90_) to characterize the cardiac AP.

### Model parameters

The stochastic parameterization yielded the model parameters (Table 3). In comparison to the parameterization of our previous model [5], the fit error of the features from the activation and deactivation protocols was reduced from 1.5 to 0.68 despite the additional error terms. Respectively the squared fit error for each feature is; C_on_: 0.06, C_off_: 0.025, A_on_: 0.11, Aoff: 0.014, t_on_: 0.04, t_off_: 0.18, I_KACh,norm,50mV_: 0.03, I_KACh,norm,-100mV_: 0.003. The total error of the features is equal to the square root of the sum of the respective squared fit errors. The corresponding modeled and measured current traces of the voltage protocols are shown in S2 Fig.

## Supporting information

S1 Fig Simulated concentration response at different holding potentials and effects of voltage shifts on the open state of the model. (A) Simulated ACh concentration-I_KACh_ response curves for voltages at indicated holding potentials. The concentration-response curves are voltage-dependent, whereby negative holding potentials are associated with greater ACh binding affinity, while depolarized holding potentials decrease binding affinity. Thus, hyperpolarizing voltage shifts in the concentration-response curve increase ACh affinity of the M2R, while depolarizing shifts decrease affinity. (B) The effects of voltage shifts (V_shift_) on the steady state of the open state of the K_ACh_ channel at 0.1μM ACh. A -30mV shift increases the O state at all voltages as a consequence of increased receptor affinity, while a +30 mV shift decreases the O state as a consequence of decreased affinity.
(TIF)

S2 Fig I_K,ACh_ current evoked by 0.1 μM ACh. Normalized simulated (A,E) and measured (B-D,F-H) [5] currents, using the activation and deactivation voltage protocols from [5]. Artifacts in the measured traces, which were cropped for the monoexponential fitting, are shown in grey.
(TIF)

S3 Fig Effect of V_*shift*_ on the open-state occupancy at a concentration of 0.08 μM ACh as well as on α and β. (A) Impact of different V_*shift*_ on the open-state occupancy, with the model being shifted in its complete depolarized/hyperpolarized state for large shifts. (B) Corresponding traces of the seperate model states over the time course of one AP. (C) Transition rates between U1 and U2 for different voltages.
(TIF)
