# Supplementary figures and images for: Modeling effects of voltage dependent properties of the cardiac muscarinic receptor on human sinus node function
Source: PLoS Comput Biol. 2018 Oct 10;14(10):e1006438. doi: 10.1371/journal.pcbi.1006438 (PMC6197694; doi:10.1371/journal.pcbi.1006438)

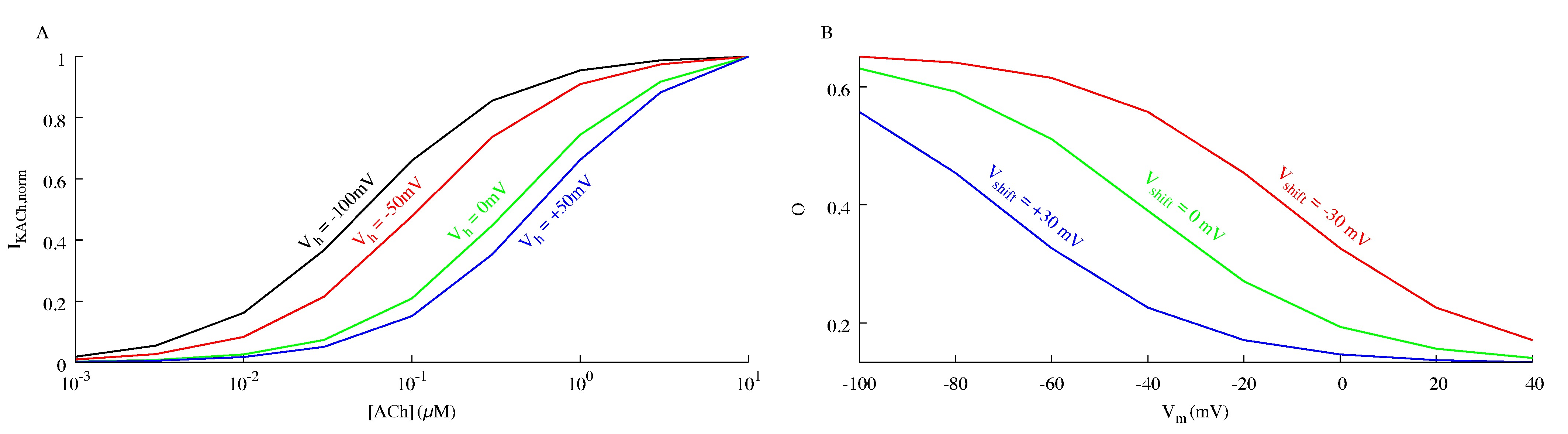

Supplement: S1 Fig — (A) Simulated ACh concentration-IKACh response curves for voltages at indicated holding potentials. The concentration-response curves are voltage-dependent, whereby negative holding potentials are associated with greater ACh binding affinity, while depolarized holding potentials decrease binding affinity. Thus, hyperpolarizing voltage shifts in the concentration-response curve increase ACh affinity of the M2R, while depolarizing shifts decrease affinity. (B) The effects of voltage shifts (Vshift) on the steady state of the open state of the KACh channel at 0.1μM ACh. A -30mV shift increases the O state at all voltages as a consequence of increased receptor affinity, while a +30 mV shift decreases the O state as a consequence of decreased affinity. (TIF) [file pcbi.1006438.s001.tif]

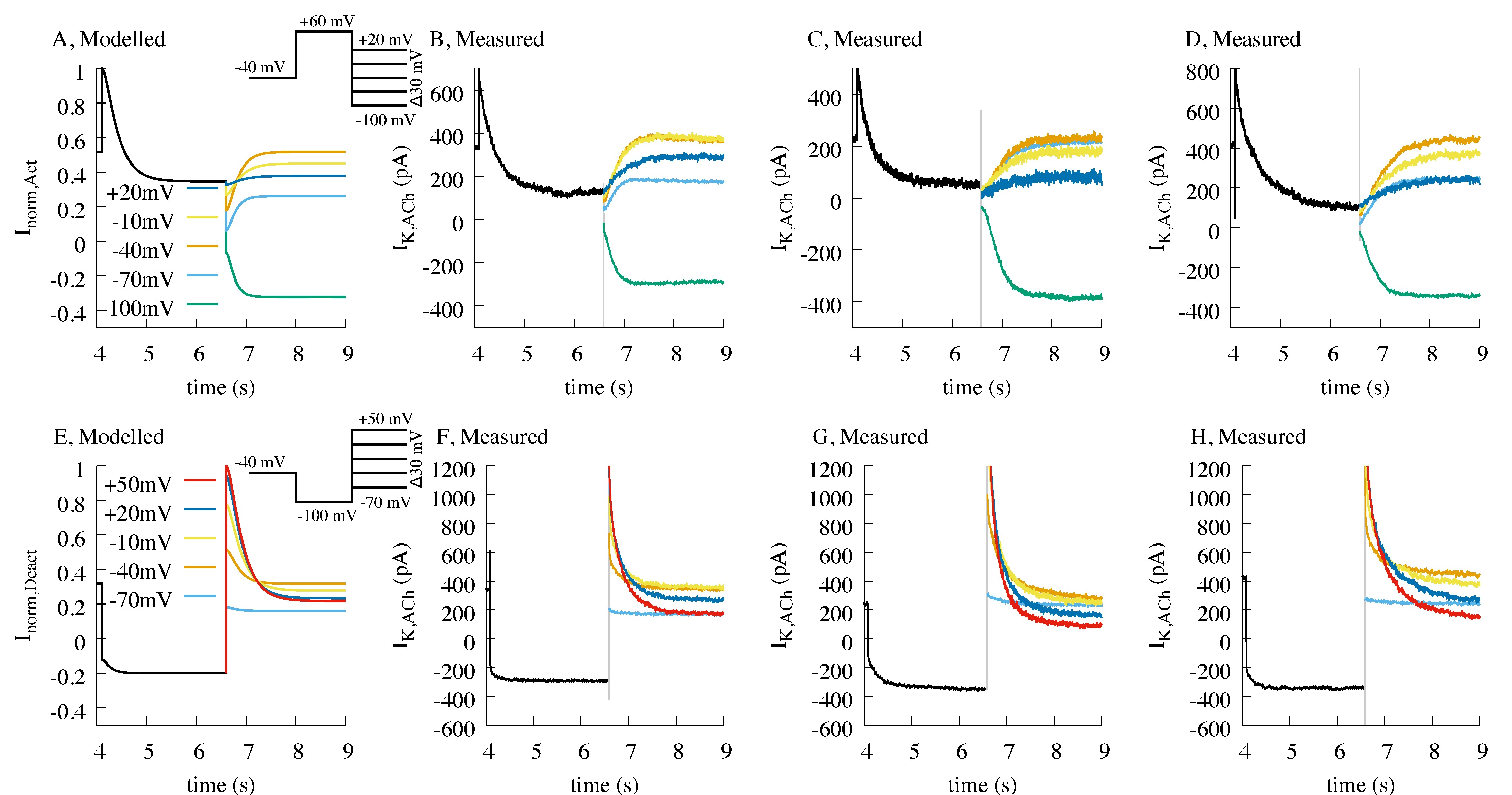

Supplement: S2 Fig — Normalized simulated (A,E) and measured (B-D,F-H) [5] currents, using the activation and deactivation voltage protocols from [5]. Artifacts in the measured traces, which were cropped for the monoexponential fitting, are shown in grey. (TIF) [file pcbi.1006438.s002.tif]

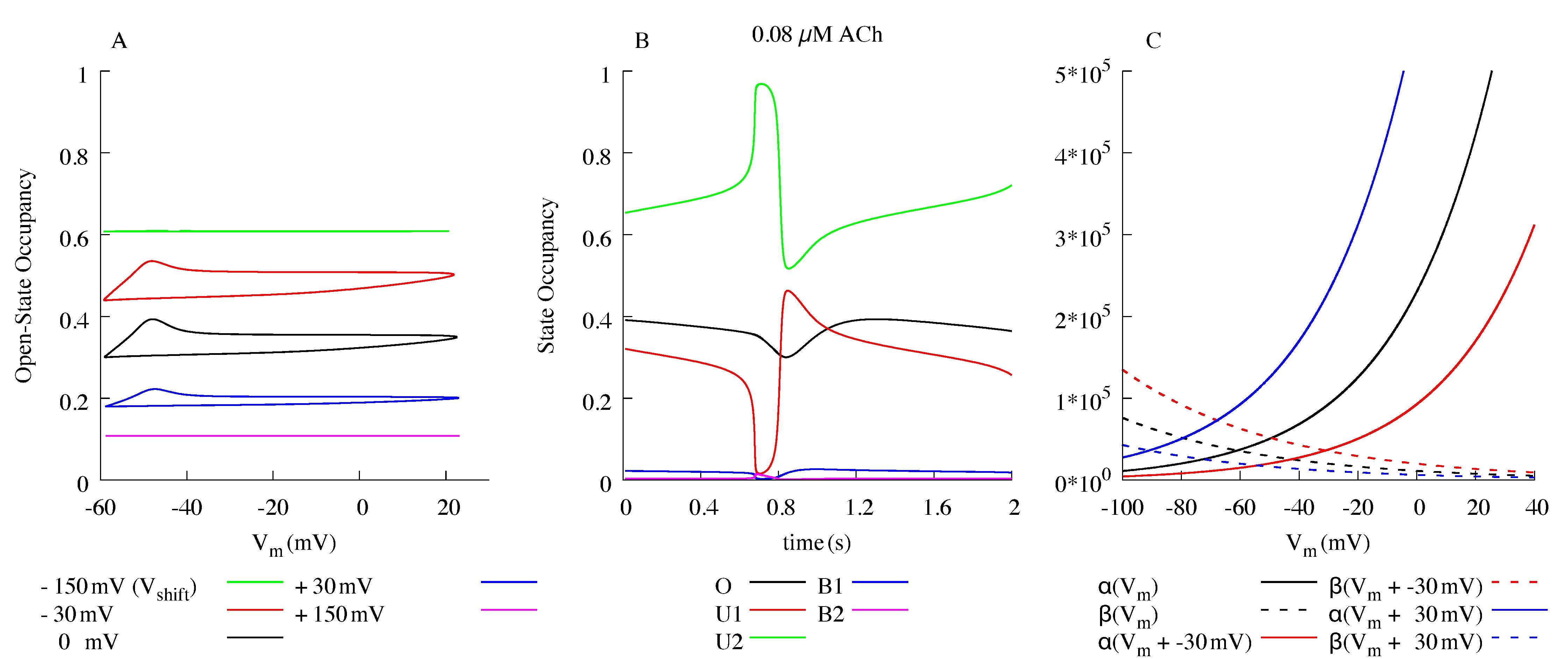

Supplement: S3 Fig — (A) Impact of different Vshift on the open-state occupancy, with the model being shifted in its complete depolarized/hyperpolarized state for large shifts. (B) Corresponding traces of the seperate model states over the time course of one AP. (C) Transition rates between U1 and U2 for different voltages. (TIF) [file pcbi.1006438.s003.tif]
